# Developing sub-acute, non-acute and special groups in the universal grouper

**DOI:** 10.1186/1472-6963-12-S1-I3

**Published:** 2012-11-21

**Authors:** Syed Mohamed Aljunid

**Affiliations:** 1 United Nations University International Institute for Global Health (UNU-IIGH), Kuala Lumpur, Malaysia

## 

The use of Casemix system as providers’ reimbursement method under social health insurance schemes has spread beyond high-income countries. Over the last decade many low and middle-income countries (LMIC) have started to implement Casemix system to replace the conventional yet inefficient fee-for-service as provider payment mechanism. UNU-IIGH launched an international grouper called UNU-CBG in 2010 to support the implementation of Casemix system in LMIC. To date, UNU-CBG has been introduced in 12 countries in different phases of implementation process. UNU-CBG was designed to function as an international grouper that can be customized and adapted to suit the local needs and norms of each country. However inability to provide robust groupings for sub-acute and chronic conditions is a major limitation in the use of Casemix system in these countries. Sub-acute and chronic diseases that account for more than two thirds of disease burden in LMIC should be categorized in mutually exclusive groups if the reimbursement were to be made using Casemix system. Researchers in UNU-IIGH and International Casemix and Clinical Coding Centre of UKM have been working very hard for the past two years to upgrade the capacity of UNU-CBG grouper to classify sub-acute and chronic cases into Casemix group. Additional variables included in the new grouping algorithm are extended length of stay, costly prostheses, drugs, procedures, investigations and scores of Activity of Daily Living. The new version of UNU-CBG launched during this conference provides an opportunity for social health insurance managers in LMIC to use Casemix system as a total solution for provider payment method.

